# Dynamic Performance Comparison of Two Kalman Filters for Rate Signal Direct Modeling and Differencing Modeling for Combining a MEMS Gyroscope Array to Improve Accuracy

**DOI:** 10.3390/s151127590

**Published:** 2015-10-30

**Authors:** Guangmin Yuan, Weizheng Yuan, Liang Xue, Jianbing Xie, Honglong Chang

**Affiliations:** 1 Ministry of Education Key Laboratory of Micro and Nano Systems for Aerospace, Northwestern Polytechnical University, No. 127 Youyi West Road, Xi’an 710072, China; E-Mails: yuangm@nwpu.edu.cn (G.Y.); xiejb@nwpu.edu.cn (J.X.); 2 Xi’an Research Institute of High Technology, Hongqing Town, Xi’an 710025, China; E-Mail: xuelmems@163.com

**Keywords:** MEMS gyroscope, Kalman filtering, sensor array, direct model, differencing model, performance comparison, optimal estimation

## Abstract

In this paper, the performance of two Kalman filter (KF) schemes based on the direct estimated model and differencing estimated model for input rate signal was thoroughly analyzed and compared for combining measurements of a sensor array to improve the accuracy of microelectromechanical system (MEMS) gyroscopes. The principles for noise reduction were presented and KF algorithms were designed to obtain the optimal rate signal estimates. The input rate signal in the direct estimated KF model was modeled with a random walk process and treated as the estimated system state. In the differencing estimated KF model, a differencing operation was established between outputs of the gyroscope array, and then the optimal estimation of input rate signal was achieved by compensating for the estimations of bias drifts for the component gyroscopes. Finally, dynamic simulations and experiments with a six-gyroscope array were implemented to compare the dynamic performance of the two KF models. The 1σ error of the gyroscopes was reduced from 1.4558°/s to 0.1203°/s by the direct estimated KF model in a constant rate test and to 0.5974°/s by the differencing estimated KF model. The estimated rate signal filtered by both models could reflect the amplitude variation of the input signal in the swing rate test and displayed a reduction factor of about three for the 1σ noise. Results illustrate that the performance of the direct estimated KF model is much higher than that of the differencing estimated KF model, with a constant input signal or lower dynamic variation. A similarity in the two KFs’ performance is observed if the input signal has a high dynamic variation.

## 1. Introduction

Microelectromechanical system (MEMS) vibratory gyroscopes are usually characterized by low cost, low power consumption, small size, and high reliability [1,2,3]. However, the low accuracy and performance of MEMS gyroscopes has restricted their development and applications to date [4]. Comprehensive research has been conducted to improve the accuracy of MEMS gyroscopes. Previous studies have effectively minimized the bias drift and measurement noise of MEMS gyroscopes. Recently, people have started to pay more attention to fusing the data from multiple gyroscopes to reduce the bias drift and improve the accuracy of MEMS gyroscopes, as multiple gyroscopes can be combined to form an array, and then a filtering technique is utilized to fuse multiple measurements together and thus achieve an optimal estimation of the rate signal, thus improving the accuracy of MEMS gyroscopes.

Numerous studies have been carried out on the multiple signal fusion of gyroscope arrays [5,6,7,8,9,10,11], which is called “virtual gyroscope” technology. Bayard and Ploen at the JPL laboratory first designed a virtual gyroscope system composed of four gyroscope arrays [5], and their simulated results demonstrated that, in a gyroscope, a 8.6604°/h drift could be greatly reduced to 0.0623°/h if the noise correlation of the sensor array approached −1/3. Previously, a gyroscope array composed of three MEMS sensors was designed [6], and a two-level Kalman filter (KF) algorithm was established to further reduce the gyroscope drift. In addition, a KF was presented to combine the outputs of six gyroscope arrays [7], in which the output of the MEMS gyroscope was described by a simplified error model to reduce the KF dimension. The effect of noise correlation on the KF performance and accuracy improvement was analyzed. Given that a gyroscope array with a reasonable correlation is difficult to design and the gyroscope array formed by multiple separate gyroscopes is usually considered to be uncorrelated [8], an approach to combine several uncorrelated MEMS gyroscopes was presented to improve accuracy. A KF for the measurement of a single MEMS gyroscope was also designed through a steady-state gain to reduce the drift and measurement noise [12], which realizes self-compensation whether in a static or dynamic environment.

The core aspect of designing a virtual gyroscope system through combining multiple gyroscopes is determining how to model the input rate signal. An analysis of the reported approaches [5,7,8,9,10,11,12] confirms that the outputs of MEMS gyroscopes are usually described as mixed measurements of the true rate signal input and random noises; in particular, the true rate signal is modeled into the KF and an optimal estimate is obtained. However, such direct model for the true rate signal has limitations that may influence the system dynamic accuracy. In the static condition, a combined rate signal with a low bias drift and the best accuracy can be obtained by the direct KF model. However, in the dynamic condition, the true rate signal and its characteristic model cannot be accurately described and obtained. Thus, a simple random walk process cannot always exactly reflect the dynamic behavior of the input rate signal. Moreover, noise variance modeling for the true rate signal cannot be accurately obtained in advance. Therefore, fusing multiple gyroscope signals for a virtual gyroscope system in the dynamic condition also lacks theoretical support and still requires further study and resolution.

To avoid directly modeling the true rate signal and solve the problem of obtaining the noise variance driving for true rate signal in the dynamic condition, a measurement differencing method was also presented for a virtual gyroscope that combines multiple gyroscopes, in which the true rate signal cannot be treated as the object of KF filtering [13]. The basic principle is that a new sequence of measurements for KF can be obtained by creating a differencing operation between the outputs of multiple gyroscopes, thus eliminating the common true rate signal included in every component gyroscopes. Afterward, the bias drifts of the component gyroscopes are modeled and estimated to compensate for the measurements of component gyroscopes in array. Nevertheless, the differencing method employs an indirect estimate scheme, and it can easily result in a low accuracy improvement because the true signal, which must be cared for and estimated, is removed.

Based on the above analysis, two significant questions worthy of discussion are raised: (1) Whether direct modeling of the true rate signal is better than the indirect differencing model in a dynamic condition; (2) Determining how to choose a suitable KF model according to the magnitude of dynamic characteristics of the input rate signal in performing a virtual gyroscope system. In this study, the direct modeling and indirect differencing modeling approaches for the true rate signal of a virtual gyroscope are investigated, and an optimal KF model with six gyroscope arrays are established. As for the direct modeling of true rate signal, the value of noise variance modeling for input rate signal is adjusted to achieve fusion of the gyroscope array outputs with different dynamic properties. Lastly, simulation and dynamic tests are performed to evaluate and compare the performance of the two KF models. The objective of this paper is to make analysis and comparison of the dynamic performance between two KF schemes. This research will provide a useful criterion for choosing a suitable model to design and implement a practical system according to the input signal dynamic variation.

## 2. Methodology Comparison of the Virtual Gyroscope System Model

### 2.1. Direct Estimated Model for Virtual Gyroscope System

The number *N* of component sensors in a gyroscope array can be selected as any integer, and from the results of [7,14], it demonstrated that the performance of a KF can be further improved through increasing number *N* of the component sensors. Additionally, the KF will show the best performance while the correlation factor *ρ* approaches −1/(*N* − 1), thus it will not need a large negative correlation factor to obtain the highest accuracy improvement by increasing the number *N*, which reduces the requirement of a negative correlation factor and is suitable for system implementation. However, the system complexity and accuracy improvement should be considered. In this study, six gyroscope arrays of *N =* 6 is selected to perform as a system and analyze the KF performance.

The MEMS gyroscope output is corrupted and influenced by several random noises [15]. Numerous experiments have shown that angular random walk (ARW) and rate random walk (RRW) are the most dominant and important random errors for MEMS gyroscopes that have low measurement precision. Consequently, in the present study, the gyroscope is described by a typical model as follows [16,17]:

(1) $$\left\{ \begin{array}{l} y(t)=\omega (t)+b(t)+n(t)\\ \dot{b}(t)=w_{b}(t) \end{array}\right.$$

where *y*(*t*) is the measured signal of the gyroscope, *ω*(*t*) is the true rate signal, *b*(*t*) is the drift driven by the RRW process *w_b_*, and *n*(*t*) is the ARW white noise. Allan variance is widely adopted to analyze the statistics of the random noise for a gyroscope [17,18,19].

As for the virtual gyroscope system modeling, to achieve an optimal estimate of the true rate signal, the true rate signal can be modeled by a random walk process and treated as the state of KF filtering [5,8]:

(2) $$\dot{\omega }=n_{\omega }$$

where *nω* is the white noise with $E[n_{\omega }(t)]=0$ and $E[n_{\omega }(t)n_{\omega }^{T}(t+\tau )]=q_{\omega }\delta (\tau )$, and *q_ω_* is the noise variance of *n_ω_*. As for a six-gyroscope array, Equation (1) can be written as [8]:

(3) $$\left\{ \begin{array}{l} Z_{1}={\left[1,1,\cdots ,1\right]}_{1\times 6}^{T}\cdot \omega +b+v_{1}\\ \dot{b}=w_{b} \end{array}\right.$$

with:

(4) $$Z_{1}={\left[y_{1},y_{2},\cdots ,y_{6}\right]}^{T},b={\left[b_{1},b_{2},\cdots ,b_{6}\right]}^{T},w_{b}={\left[w_{b1},w_{b1},\cdots ,w_{b6}\right]}^{T},v_{1}={\left[n_{1},n_{2},\cdots ,n_{6}\right]}^{T}$$

where $y_{i}$ is the output of the *i*th gyroscope, $Z_{1}(t)$ is the measurement values of the gyroscope array, $b_{i}$ is the bias drift of the *i*th gyroscope, $w_{bi}$ is the RRW noise of the *i*th gyroscope, and $n_{i}$ is the ARW of the *i*th gyroscope.

The true rate signal *ω* and bias drifts of the component gyroscopes ***b*** are set as the system estimated quantity to make optimal estimations. Using KF method [20], and on the basis of the Equations (2) and (3), the filtering state-space model for a virtual gyroscope can be formed as follows:

(5) $$\left\{ \begin{array}{l} X_{1}(t)={\left[b,\omega \right]}^{T}\\ {\dot{X}}_{1}(t)=F_{1}\cdot X_{1}(t)+w_{1}(t)\\ Z_{1}(t)=H_{1}\cdot X_{1}(t)+v_{1}(t) \end{array}\right.$$

The KF coefficient matrices $F_{1}=0_{7\times 7}$ and $H_{1}$ are given as:

(6) $$H_{1}={\left[\begin{array}{cccccc} 1 & 0 & 0 & \cdots & 0 & 1\\ 0 & 1 & 0 & \cdots & 0 & 1\\ 0 & 0 & 1 & \cdots & 0 & 1\\ \vdots & \vdots & \vdots & \ddots & \vdots & \vdots \\ 0 & 0 & 0 & \cdots & 1 & 1 \end{array}\right]}_{6\times 7}$$

In Equation (5), ***w***_1_(*t*)*=*[***w****_b_*,*n_ω_*]*^T^* is the system process noise vector and ***v***_1_(*t*) is the measurement noise vector with the variance as:

(7) $$\left\{ \begin{array}{l} E\left[w_{1}(t)\right]=0,E\left[w_{1}(t)w_{1}^{T}(t+\tau )\right]=Q_{1}\delta (\tau )\\ E\left[v_{1}(t)\right]=0,E[v_{1}(t)v_{1}^{T}(t+\tau )]=R_{1}\delta (\tau )\\ E\left[w_{b}(t)\right]=0,E[w_{b}(t)w_{b}^{T}(t+\tau )]=Q_{b}\delta (\tau ) \end{array}\right.,Q_{1}=\left[\begin{array}{l} Q_{b}\;0\\ 0\;q_{\omega } \end{array}\right]$$

Based on the state-space model of Equation (5), the continuous-time KF for obtaining the optimal estimates of the true rate signal and bias drifts can be given as follows:

(8) $$\left\{ \begin{array}{l} {\dot{\hat{X}}}_{1}(t)=K_{1}(t)\left[Z_{1}(t)-H_{1}{\hat{X}}_{1}(t)\right]\\ K_{1}(t)=P_{1}(t)H_{1}^{T}R_{1}^{-1}\\ {\dot{P}}_{1}(t)=Q_{1}-P_{1}(t)H_{1}^{T}R_{1}^{-1}H_{1}P_{1}(t) \end{array}\right.$$

where ${\hat{X}}_{1}(t)$ is the estimation of $X_{1}(t)$, $K_{1}(t)$ is the filtering gain, and $P_{1}(t)$ is the filtering estimated covariance. To explain the accuracy improvement and discover the inherent system properties in a virtual gyroscope system, Bayard and Ploen employed an analytic approach to provide a solution to the continuous-time KF of Equation (8) in [5] and thus obtained a steady filtering gain. Therefore, the discrete-time KF for estimating the true rate signal can be formed as follows [5,8]:

(9) $$\left\{ \begin{array}{l} {\hat{\zeta }}_{k+1}=[\begin{array}{cc} e^{-{\bar{D}}^{\frac{1}{2}}}T & 0\\ 0 & 1 \end{array}]{\hat{\zeta }}_{k}+S^{-1}\bar{K}Z_{k}\\ {\hat{\omega }}_{k}=e_{7}^{T}S{\hat{\zeta }}_{k} \end{array}\right.$$

where *T* is the sampling period, $\bar{K}$ is the filtering gain matrix, $\hat{\zeta }$ is the discrete-time KF state vector, $\bar{D}$ is a diagonal matrix composed of the nonzero eigenvalues, ${\hat{\omega }}_{k}$ is the true rate signal estimate, and vector $e_{7}={\left[0,0,\ldots 0,1\right]}^{T}$. The concrete definition of the matrices $S,\;\bar{D},\;K_{\infty },\bar{K}$ can be referred and obtained from [5].

From the above virtual gyroscope model, the input true rate signal is directly modeled by a random walk process with a noise variance *q_ω_* and set into the system state. Thus, the performance of the virtual gyroscope and KF are heavily related to the value of *q_ω_*. Essentially, the parameter *q_ω_* reflects the magnitude of the dynamic behavior of the input rate signal. In practice, choosing a value of *q_ω_* should be matched with the dynamic behavior of the input rate signal. Thus, if the input signal has a small dynamic variation, it should select a smaller value of the *q_ω_* to perform the KF and obtain a higher accuracy. If the input signal has a high dynamic behavior, a larger *q_ω_* value should be chosen to ensure that the KF results in smaller amplitude attenuation for the original signal, where the term “attenuation” implies that the amplitude of the estimated rate signal is smaller than that of the input signal in a dynamic condition. In this sense, the bandwidth of KF mainly depends on the value of *q_ω_*. Therefore, the true rate signal can be well estimated through adjusting the *q_ω_*, and thus improving the dynamic accuracy of KF. In the subsequent section, the relationship of the parameter *q_ω_* with KF bandwidth will be further analyzed, and the *q_ω_* will be adjusted to process the outputs of the gyroscope array in simulation and experiment.

### 2.2. Differencing Estimated Model for the Virtual Gyroscope System

In the static condition, the gyroscope true rate signal can be regarded as zero. In this study, the differencing estimated model is designed for the virtual gyroscope system in a dynamic condition. The bias drift *b_i_* of the component gyroscopes taken as the filtered object of KF, and the gyroscope error model of Equation (1) is used to form the system state equation as follows [13]:

(10) $$\left\{ \begin{array}{l} X_{2}(t)={\left[b1,b2,b3,b4,b5,b6\right]}^{T}\\ {\dot{X}}_{2}(t)=\dot{b}=w_{b} \end{array}\right.$$

where $w_{b}={\left[w_{b1},w_{b1},\cdots ,w_{b6}\right]}^{T}$. A specific differencing method is then adopted to solve the problem of modeling the true rate signal. The mutual subtraction of the multiple gyroscope outputs between every two gyroscopes in the array can eliminate the true rate signal *ω*. The corresponding equation for the measurement of KF can be formed as [13]:

(11) $$\left[\begin{array}{c} y_{2}-y_{1}\\ y_{3}-y_{2}\\ y_{4}-y_{3}\\ \vdots \\ y_{1}-y_{6} \end{array}\right]=\left[\begin{array}{ccccc} -1 & 1 & 0 & \cdots & 0\\ 0 & -1 & 1 & \cdots & 0\\ 0 & 0 & -1 & \cdots & 0\\ \vdots & \vdots & \vdots & \ddots & \vdots \\ 1 & 0 & 0 & \cdots & -1 \end{array}\right]\cdot X_{2}(t)+\left[\begin{array}{c} n_{2}-n_{1}\\ n_{3}-n_{2}\\ n_{4}-n_{3}\\ \vdots \\ n_{1}-n_{6} \end{array}\right]$$

Based on Equations (10) and (11), the virtual gyroscope KF state-space model for the differencing estimated model can be given as:

(12) $$\left\{ \begin{array}{l} {\dot{X}}_{2}(t)=F_{2}\cdot X_{2}(t)+w_{2}(t)\\ Z_{2}(t)=H_{2}\cdot X_{2}(t)+v_{2}(t) \end{array}\right.$$

with:

(13) $$Z_{2}(t)=\left[\begin{array}{c} y_{2}-y_{1}\\ y_{3}-y_{2}\\ y_{4}-y_{3}\\ \vdots \\ y_{1}-y_{6} \end{array}\right],F_{2}=0_{6\times 6},w_{2}(t)=\left[\begin{array}{c} w_{b1}\\ w_{b2}\\ \vdots \\ w_{b6} \end{array}\right],v_{2}(t)=\left[\begin{array}{c} n_{2}-n_{1}\\ n_{3}-n_{2}\\ \vdots \\ n_{1}-n_{6} \end{array}\right],H_{2}=\left[\begin{array}{ccccc} -1 & 1 & 0 & \cdots & 0\\ 0 & -1 & 1 & \cdots & 0\\ 0 & 0 & -1 & \cdots & 0\\ \vdots & \vdots & \vdots & \ddots & \vdots \\ 1 & 0 & 0 & \cdots & -1 \end{array}\right]$$

where $Z_{2}(t)$ is the differencing measurements of the sensor array, $F_{2}$ and $H_{2}$ are the KF coefficient matrices, and $w_{2}(t)$ and $v_{2}(t)$ are white noise vectors that represent the KF process noise and measurement noise respectively. Given that no correlation exists between the ARW noises *n_i_* of the component gyroscopes, and $\sigma_{ni}^{2}$ is the variance for ARW of the *i*th gyroscope, the measurement covariance matrix $R_{2}$ of vector $v_{2}(t)$ can be formed as follows:

(14) $$R_{2}=\left[\begin{array}{ccccc} \sigma_{n1}^{2}+\sigma_{n2}^{2} & -\sigma_{n2}^{2} & 0 & \cdots & -\sigma_{n1}^{2}\\ -\sigma_{n2}^{2} & \sigma_{n2}^{2}+\sigma_{n3}^{2} & -\sigma_{n3}^{2} & \cdots & 0\\ 0 & -\sigma_{n3}^{2} & \sigma_{n3}^{2}+\sigma_{n4}^{2} & \cdots & 0\\ \vdots & \vdots & \vdots & \ddots & \vdots \\ -\sigma_{n1}^{2} & 0 & 0 & \cdots & \sigma_{n1}^{2}+\sigma_{n6}^{2} \end{array}\right]$$

**Figure 1:**
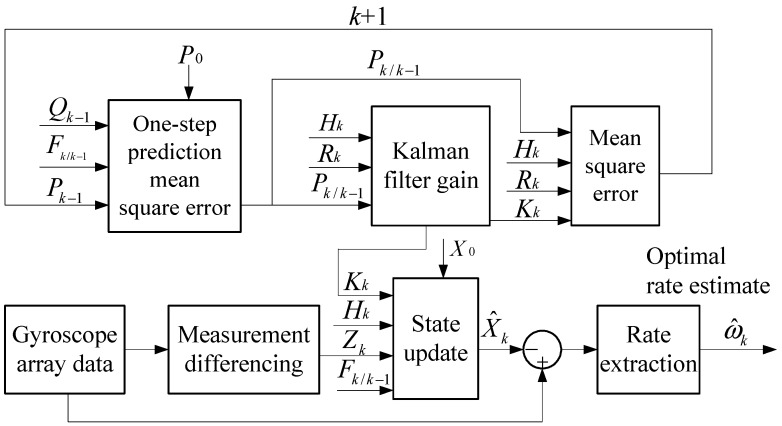
Principle block diagram of the differencing KF model.

Discretization of the continuous-time KF of Equation (12), which is applied here as a basic discrete iterative KF method as shown in Equations (15)–(18), is used to implement the filter [20]. Consequently, the gyroscope bias drifts ***b*** can be estimated in the following discrete iterative equations, and then the outputs of the component gyroscopes can be compensated by the bias drift estimate. The principle block diagram of the differencing KF model is shown in Figure 1:

(15) $$P_{k/k-1}=F_{k,k-1}P_{k-1}F_{k,k-1}^{T}+Q_{k-1}$$

(16) $$K_{k}=P_{k/k-1}H_{k}^{T}{\left(H_{k}P_{k/k-1}H_{k}^{T}+R_{k}\right)}^{-1}$$

(17) $$P_{k}=(I-K_{k}H_{k})P_{k/k-1}{\left(I-K_{k}H_{k}\right)}^{T}+K_{k}R_{k}K_{k}^{T}$$

(18) $${\hat{X}}_{k}=F_{k,k-1}{\hat{X}}_{k-1}+K_{k}(Z_{k}-H_{k}F_{k,k-1}{\hat{X}}_{k-1})$$

Thus, the combined rate signal can be obtained by calculating the arithmetic average of the compensated component gyroscopes as:

(19) $${\hat{\omega }}_{k}=\sum_{i=1}^{N}\frac{1}{N}(y_{i,k}-{\hat{b}}_{i,k})$$

## 3. Bandwidth Analysis of the Two KF Models

For the direct estimated KF model, the true rate signal is directly modeled by a random walk driven by a white noise with the variance *q_ω_* and set into the system estimated state. Thus, the KF bandwidth mainly depends on the term *q_ω_*. In this study, we utilize the KF frequency response to analyze the KF bandwidth, and attempt to establish a suitable model to represent the relationship of the KF bandwidth and *q_ω_*.

As for the direct estimated model, based on the continuous-time KF of Equation (5), the transfer relationship from the gyroscope array measurements to the virtual gyroscope output can be shown in the Laplace transform domain as follows [8]:

(20) $$\hat{\omega }(s)=e_{7}^{T}{\left(sI+K_{\infty }H_{1}\right)}^{-1}K_{\infty }Z_{1}(s)$$

Thus, the transform function can be expressed as:

(21) $$H(s)=e_{7}^{T}{\left(sI+K_{\infty }H_{1}\right)}^{-1}K_{\infty }*{\left[1,1,\ldots 1\right]}_{1\times 6}^{T}$$

**Figure 2:**
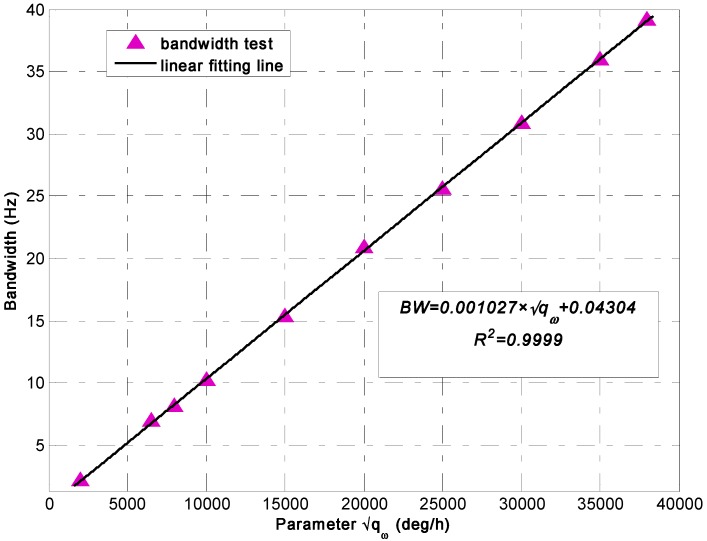
Linear fitting result of the KF −3 dB bandwidth with parameter $\sqrt{q_{\omega }}$.

When Equation (21) is used and the 95% confidence interval is taken, a linear model is obtained to represent the relationship of the −3 dB bandwidth of KF and *q_ω_* (see in Figure 2). The fitting result is shown in Table 1.

(22) $$BW=0.001027\times \sqrt{q_{\omega }}+\text{0}.\text{04304}$$

Table 1 shows that the fitting error of root mean square error (RMSE) is approximately 0.1566 and sum of square error (SSE) is 0.1961; thus, the linear model can be used to well represent the relationship of the KF bandwidth with $\sqrt{q_{\omega }}$. The KF bandwidth will be considerably higher than that of the individual gyroscope when the parameter *q_ω_* is set to a very large value. Therefore, the bandwidth of the entire virtual gyroscope system will be comparable to that of the individual gyroscope.

Based on the established differencing estimated model derived from Equation (19) in Section 2.2, the bandwidth of the virtual gyroscope system is comparable to that of the component gyroscopes, in that only the bias drifts of sensor arrays are modeled and set into the KF estimated quantity.

**Table 1:** Fitting result of the KF bandwidth *vs.* parameter *q_ω_* ($BW=P_{1}\times \sqrt{q_{\omega }}+P_{2}$).

Coefficient	*P_1_*	*P_2_*	SSE	RMSE	R^2^
Value	0.001027	0.04304	0.1961	0.1566	0.9999
Bound	(0.001018, 0.001037)	(–0.1704, 0.2565)			

The above analysis confirmed that the KF bandwidth for the direct estimated model can be intentionally adjusted by means of choosing different *q_ω_* values. Thus, the bandwidth of the virtual gyroscope system will either be smaller than or comparable to that of the component gyroscopes because a complete virtual gyroscope system is composed of two parts, namely, a sensor array and a KF algorithm processor. On the contrary, the KF bandwidth for the differencing estimated model cannot be adjusted, and the virtual gyroscope system bandwidth is comparable to that of the component gyroscopes. Therefore, the KF performance for the direct estimated model will be higher than that of the differencing estimated model through decreasing the KF bandwidth when the input rate signal has a small dynamic variation.

## 4. Dynamic Simulation Comparison of Two KF Models

**Figure 3:**
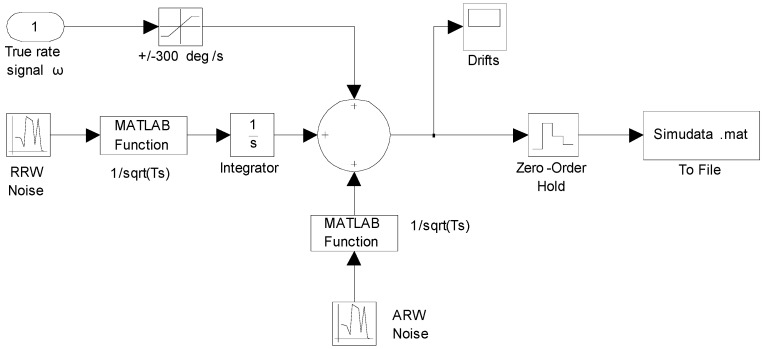
Simulink model for generating outputs of the gyroscope array signals.

No correlation of ARW noises with RRW noises of the component sensors exists. Hence, various dynamic simulations of the constant input rate and sinusoidal input rate signal based on a six-gyroscope array were carried out to verify the performance of the two KF models. The output signals of the gyroscope array were simulated by the gyroscope model of Equation (1). The Simulink model is shown in Figure 3.

The standard deviation (STD, 1σ) of the rate errors is adopted to quantify the measurement precision of the rate signal in the dynamic condition. The mathematical expression of the STD can be defined as:

(23) $$\text{σ}=\sqrt{\frac{1}{n-1}\sum_{k=1}^{n}{\left({\hat{\omega }}_{k}-\omega_{k,true}\right)}^{2}}$$

where $\omega_{k,true}$ is the true rate signal of the *k*th time, ${\hat{\omega }}_{k}$ is the estimation of $\omega_{k,true}$, and *n* is the length number of rate samples.

**Figure 4:**
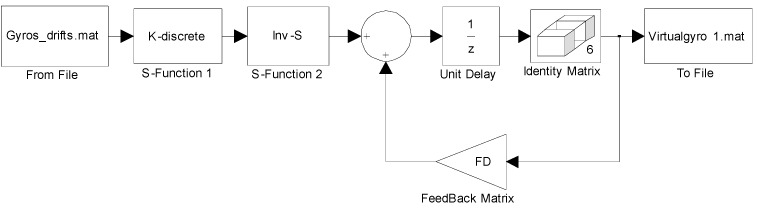
Simulink model for discrete-time KF of the direct estimated model.

The algorithm of the direct estimated model described by Equation (9) in Simulink is shown in Figure 4. Concretely, the block of “Gyros_drifts.mat” in Figure 4 refers to $Z_{k}$ in Equation (9), the blocks of “K-discrete”, “Inv-S” and “FD” refer to $\bar{K}$, $S^{-1}$, and $\left[\begin{array}{cc} e^{-{\bar{D}}^{\frac{1}{2}}}T & 0\\ 0 & 1 \end{array}\right]$ respectively in Equation (9).

### 4.1. Constant Rate Simulation Result

**Figure 5:**
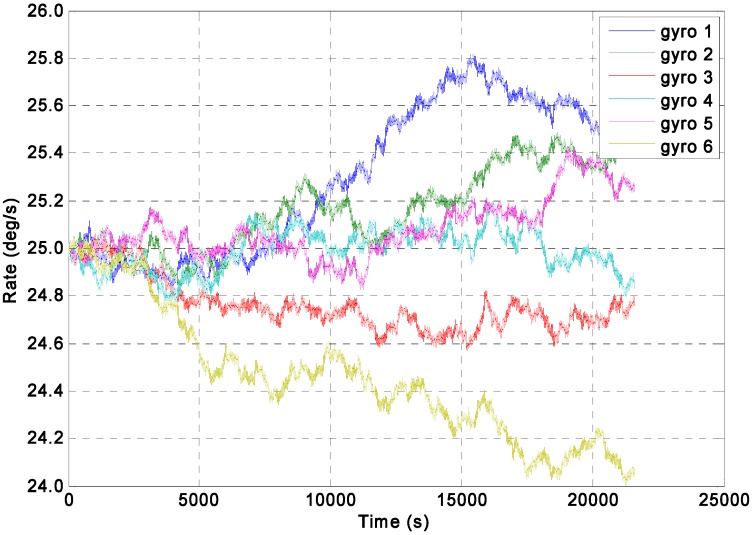
Dynamic simulation of the gyroscope array signals with a constant input rate signal of *ω =* 25°/s.

**Figure 6:**
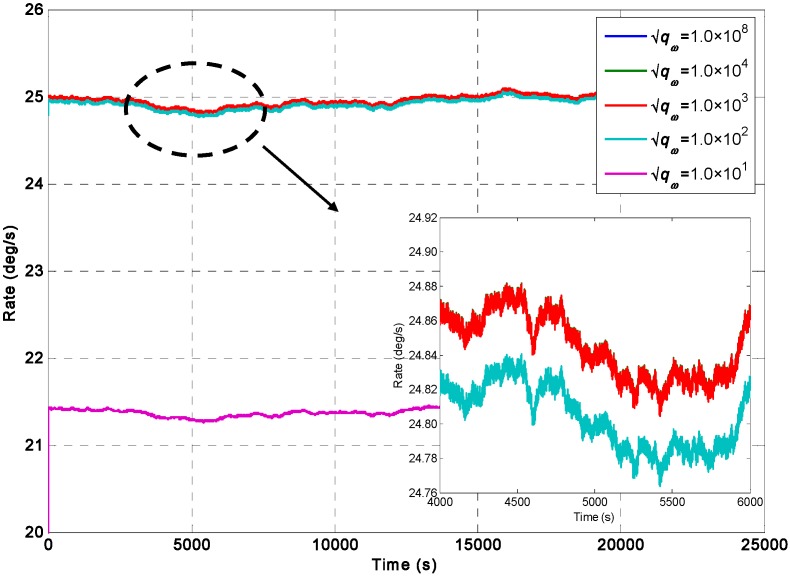
Filtering results of the direct estimated KF model with a constant input rate signal of *ω =* 25°/s under different values of $\sqrt{q_{\omega }}$.

**Figure 7:**
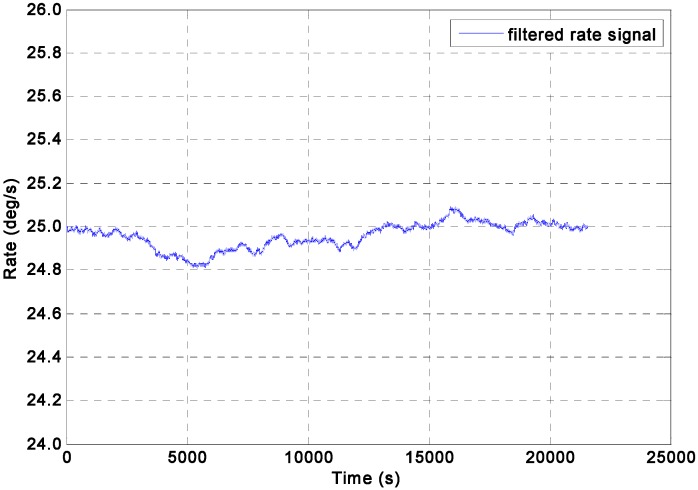
Rate signals filtered by the differencing estimated KF model with a constant input rate signal of *ω =* 25°/s.

For the constant rate simulation, a constant input rate of *ω =* 25°/s is given to evaluate the performance of the two KF models. The output signals of the gyroscope array are generated by the Simulink model shown in Figure 3, where the ARW and RRW noises for the component gyroscopes are assumed to be 0.0833°/h^0.5^ and 600°/h/h^0.5^ respectively. The $\sqrt{q_{\omega }}$ is chosen with different magnitude values of 1.0 × 10^8^, 1.0 × 10^4^, 1.0 × 10^3^, 1.0 × 10^2^, and 10°/h, respectively, for the direct estimated KF model. The filtering results of the constant input rate signal using the Simulink model (Figure 4) are shown in Figure 5 and Figure 6. The combined rate signal obtained by the differencing estimated KF model is shown in Figure 7. The compared results of 1σ error are illustrated in Table 2.

The input rate signal in the direct estimated KF model can be well estimated, as shown in Figure 6. Likewise, Table 2 illustrates that the mean value of the estimated rate signal approached 25°/s when the value of $\sqrt{q_{\omega }}$ was larger than 10°/h; in particular, the 1σ error of the individual gyroscopes at 0.1797°/s was decreased by the KF to 0.0592°/s, which reached a reduction factor of about 3.03 for the dynamic noise. Table 2 also shows that the different values of *q_ω_* for the KF did not significantly affect the noise reduction and accuracy improvement when the $\sqrt{q_{\omega }}$ was larger than 10°/h. Therefore, a smaller *q_ω_* value can be chosen as the constant input rate signal. The findings also indicated that the performance of the direct estimated KF model was better than that of the differencing estimated KF model for a constant input rate signal.

**Table 2:** Performance comparison of two virtual gyroscope KF models with a constant input rate signal (*ω =* 25°/s).

Virtual Gyroscope KF Model	$\sqrt{q_{\omega }}$ (°/h)	Mean of Estimated Rate Signal (°/s)	STD of Estimated Error (1σ, °/s)
Direct Estimated Model	1.0 × 10^8^	24.9576	0.0594
	1.0 × 10^4^	24.9576	0.0592
	1.0 × 10^3^	24.9572	0.0590
	1.0 × 10^2^	24.9161	0.0591
	10	21.3921	0.0613
Differencing Estimated Model	—	24.9576	0.0613

It should be noted that the 1σ values of estimated error for direct KF model are smaller than that for indirect differencing KF model, but their difference are very small, with only about 8.3°/h. The reason could be analyzed as follows: as for the direct KF model, the KF performance would be higher than that of the indirect KF model through setting the *q_ω_* to a smaller value to reduce the KF bandwidth under condition of a constant rate signal input. However, the approach of reducing bandwidth to improve accuracy and obtain a significant noise reduction is more obvious for ARW noise rather than RRW, it can be said that the noise reduction for the ARW will be greater than that for RRW. However, from Figure 5, it can be seen that the RRW is the dominant noise compared to the ARW in the component gyroscopes. Thus the noise characteristic in the component gyroscopes (Figure 5) would result in a small difference between two KF models.

On the other hand, as illustrated in Table 2, the values of mean and 1σ error for indirect differencing KF model are close to the direct KF model with $\sqrt{q_{\omega }}$ = 1.0 × 10^8^°/h, but the 1σ error reached to 0.0591°/s while reducing the parameter *q_ω_* to $\sqrt{q_{\omega }}$ = 100°/h, which is smaller than that for differencing KF model. Thus, as for the direct KF model, if the input signal has a constant property or small dynamic variation, the parameter *q_ω_* can be set to a small value to reduce the KF bandwidth to obtain a higher accuracy improvement.

### 4.2. Sinusoidal Rate Simulation Result

**Figure 8:**
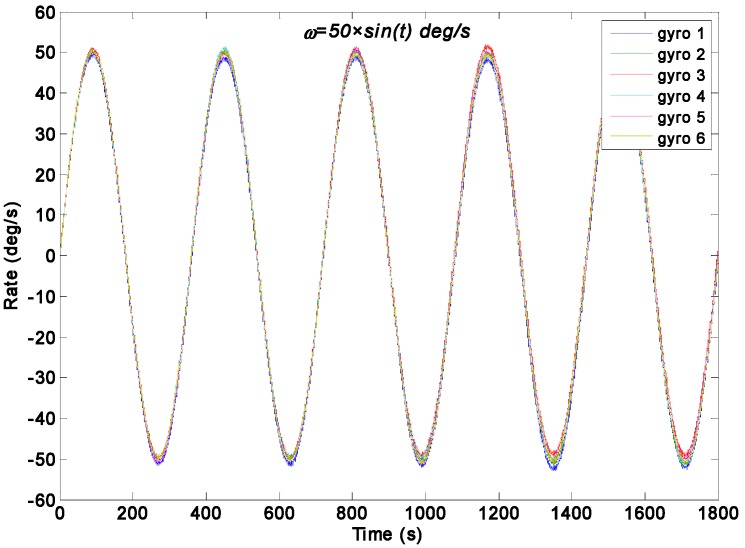
Dynamic simulation of the gyroscope array signals with a sinusoidal input rate signal of *ω =* 50 × *sin*(*t*)°/s.

**Figure 9:**
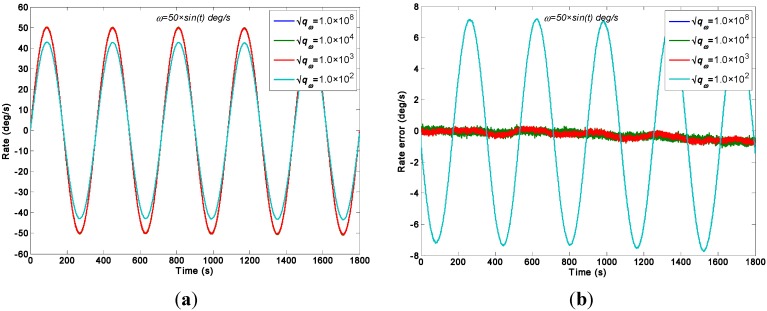
Filtering results of the direct estimated KF model with different $\sqrt{q_{\omega }}$ values of a sinusoidal input rate signal [*ω =* 50 × *sin*(*t*)°/s]. (**a**) Estimated rate signal; (**b**) Estimated errors.

**Figure 10:**
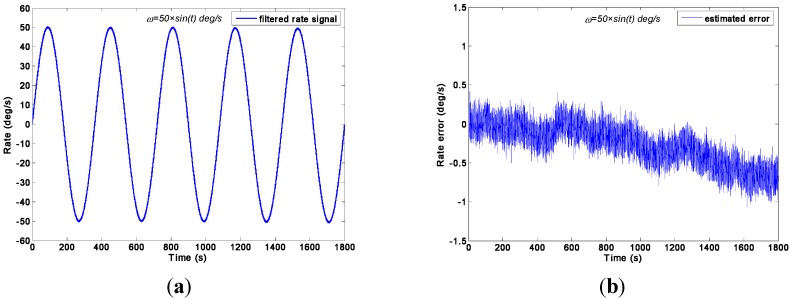
Virtual gyroscope results by the differencing estimated KF model of a sinusoidal input rate signal [*ω =* 50 × *sin*(*t*)°/s]. (**a**) Estimated rate signal; (**b**) Estimated errors.

As for the sinusoidal rate simulation, the input rate signal is given by a sinusoidal signal as *ω =* 50 × *sin*(*t*)°/s with frequency *f =* 0.1592 Hz and phase *φ_0_ =* 0. The output signals of the gyroscope array are generated by the Simulink model (Figure 3). The ARW and RRW noises are assumed to be 5.0°/h^0.5^ and 6000°/h/h^0.5^ for the component gyroscopes respectively. The parameter $\sqrt{q_{\omega }}$ is chosen for different values of 1.0 × 10^8^, 1.0 × 10^4^, 1.0 × 10^3^, and 1.0 × 10^2^°/h for the direct estimated KF model. Figure 8 and Figure 9 show the filtering results when the Simulink model (Figure 4) was used. The estimated rate signals and errors by the differencing KF model are shown in Figure 10. The compared results are shown in Table 3.

**Table 3:** Performance comparison of two virtual gyroscope KF models with a sinusoidal input signal rate (*ω =* 50 × *sin*(*t*)°/s).

Virtual Gyroscope KF Model	$\sqrt{q_{\omega }}$ (°/h)	Amplitude of Rate Signal (°/s)	STD of Estimated Error (1σ, °/s)
Direct Estimated Model	1.0 × 10^8^	50.2677	0.2506
	1.0 × 10^4^	50.2668	0.2505
	1.0 × 10^3^	50.1195	0.2480
	1.0 × 10^2^	42.9318	5.0860
Differencing Estimated Model	—	50.2677	0.2506
Original Individual Gyroscope	—	51.1120	0.5072

For the direct estimated KF model, Figure 9 shows that the estimated rate signal can reflect and reproduce the dynamic characteristic of the input rate signal when the parameter $\sqrt{q_{\omega }}$ chosen is 1.0 × 10^8^, 1.0 × 10^4^ and 1.0 × 10^3^°/h, and the measurement noise is reduced. However, when the value of $\sqrt{q_{\omega }}$ is smaller than 100°/h, it results in a larger signal attenuation for the signal amplitude. Such attenuation is attributed to the choice of $\sqrt{q_{\omega }}$ because a value of 100°/h cannot match the dynamic characteristic of the input rate signal. Additionally, Figure 10 shows that the estimated rate signal by the differencing estimated KF model can reflect the dynamic property of the input rate signal without attenuation.

As indicated in Table 3, the amplitudes of estimated rate signal by the two different KF models nearly reached 50.1°/s, which fit the simulated setting of 50°/s without a larger attenuation. Especially, in Table 3, it can be seen that the KF resulted in a larger attenuation, and the amplitude of estimated rate signal only reached to about 42.93°/s, which is lower than that of the original gyroscope at 50°/s, eventually leading to a STD of 5.086°/s for estimated error, which is larger than that of the rest. Meanwhile, the 1σ error of the individual gyroscopes at 0.5072°/s was reduced to 0.2480°/s, which achieved a reduction factor of about 2.0 for the dynamic noise. In particular, this result demonstrates that the reduction of noise and the performances of both KF models are comparable in such sinusoidal input rate simulation. This finding is attributed to the large value of parameter *q_ω_* of the direct estimated KF model, a value that ensures the KF bandwidth will reflect the dynamic proper of the input rate signal, and thus guarantee the estimated rate signal without amplitude attenuation. Based on the results, it illustrates that the feasibility and effectiveness of the direct estimated KF model will be low while reducing the KF bandwidth to obtain a remarkable noise reduction in a high dynamic application.

## 5. Dynamic Experiment Comparison and Discussion

**Figure 11:**
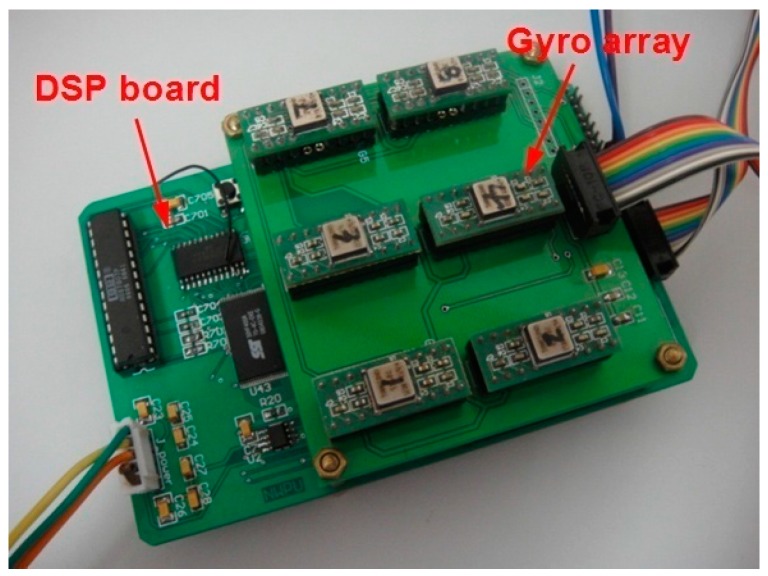
Prototype of the designed virtual gyroscope system with six component sensors.

Six ADXRS300 gyroscopes [21] were used to form a sensor array for the virtual gyroscope system with constant input rate and swing input rate tests (Figure 11).The STD of the estimated errors was also used to evaluate the accuracy of the gyroscope rate signal in the dynamic condition.

### 5.1. Constant Rate Signal Test Result

**Figure 12:**
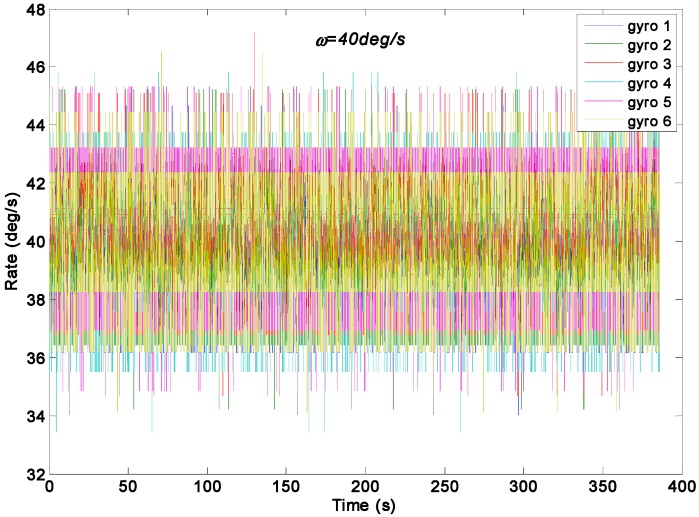
Outputs of the gyroscope array with a constant input rate signal of *ω =* 40°/s.

**Figure 13:**
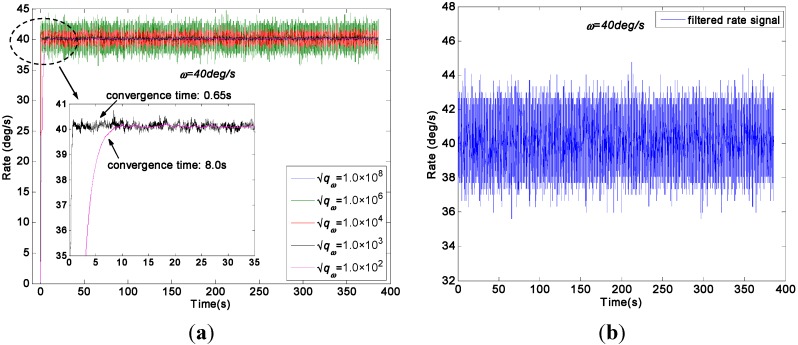
Virtual gyroscope test results of two KF models with a constant input rate signal of *ω =* 40°/s. (**a**) Estimated rate signal by the direct KF model; (**b**) Estimated rate signal by the differencing KF model.

A constant input rate signal of *ω =* 40°/s was used to test the two KF models on a turntable. The original gyroscope signals are shown in Figure 12. The outputs of the virtual gyroscope system filtered by two KF models are shown in Figure 13, where the parameter $\sqrt{q_{\omega }}$ was chosen as 1.0 × 10^8^, 1.0 × 10^6^, 1.0 × 10^4^, 1.0 × 10^3^, and 1.0 × 10^2^°/h. The STD results are given in Table 4.

Figure 13a indicates that the measurement noise can be significantly decreased by the direct estimated KF model, while the parameter $\sqrt{q_{\omega }}$ was chosen as 1000 and 100°/h. This finding can be attributed to the very small dynamic variation of input signals because of the constant rate test condition, which well fits the KF model. Figure 13b shows that noise were also reduced by the differencing estimated KF model; however, its reduction magnitude is smaller than that of the direct estimated KF model. In particular, Figure 13a displays a convergent region and convergence time for the estimated rate signal while the values of $\sqrt{q_{\omega }}$ was chosen within a small range. Furthermore, the convergence time was about 0.65 s and 8.0 s for the rate signal with a choice of $\sqrt{q_{\omega }}$ = 1000 and 100°/h respectively, which indicate an increase of convergence time with decreasing $\sqrt{q_{\omega }}$ value.

**Table 4:** Compared results of two virtual gyroscope KF models with a constant input rate signal test (*ω =* 40°/s).

Virtual gyroscope KF model	$\sqrt{q_{\omega }}$ (°/h)	Mean of Estimated Signal (°/s)	STD of Rate Error (1σ, °/s)
Direct Estimated Model	1.0 × 10^8^	40.1457	0.5960
	1.0 × 10^6^	40.1457	0.5857
	1.0 × 10^4^	40.1445	0.5235
	1.0 × 10^3^	40.1301	0.1203
	1.0 × 10^2^	39.9752	0.0832
Differencing Estimated Model	—	40.1457	0.5974
Original Individual Gyroscope	—	40.2457	1.4558

As shown in Table 4, the mean of estimated rate signal of the two KF models is about of 40.1°/s. The 1σ error of the individual gyroscope at approximately 1.4558°/s was reduced to 0.1203°/s and 0.0832°/s by the direct estimated KF model with choice of $\sqrt{q_{\omega }}$ = 1000 and 100°/h, respectively, which resulted in reduction factors of about 12.1 and 17.5. By contrast, the 1σ error was reduced to 0.5974°/s by the differencing estimated KF model. Thus, the findings confirmed that the performance of the direct estimated KF model is higher than that of the differencing estimated KF model under the input rate signal with a small dynamic variation because the KF bandwidth of direct estimated model can be adjusted. Therefore, an estimated rate signal with a low drift and noise level could be obtained by adjusting the parameter *q_ω_* with consideration of the tradeoff accuracy for convergence time.

### 5.2. Swing Rate Signal Test Result

**Figure 14:**
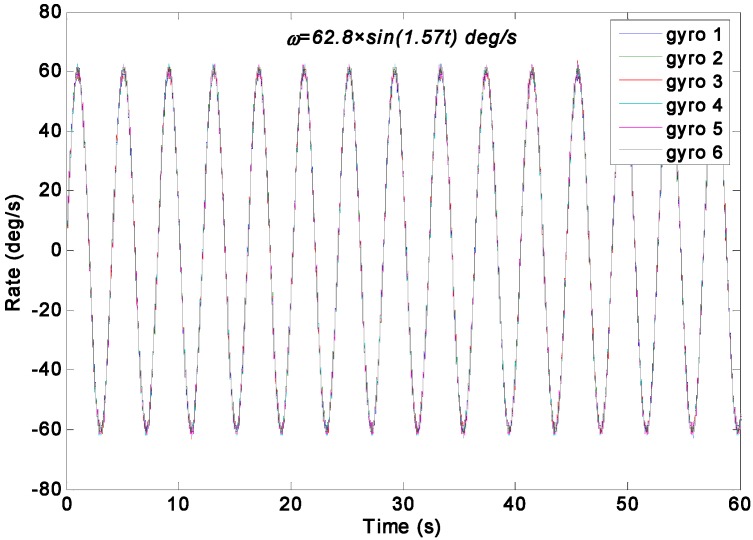
Output signals of the gyroscope array with a sinusoidal input signal of *ω =* 62.8 × *sin*(1.57*t*)°/s.

**Figure 15:**
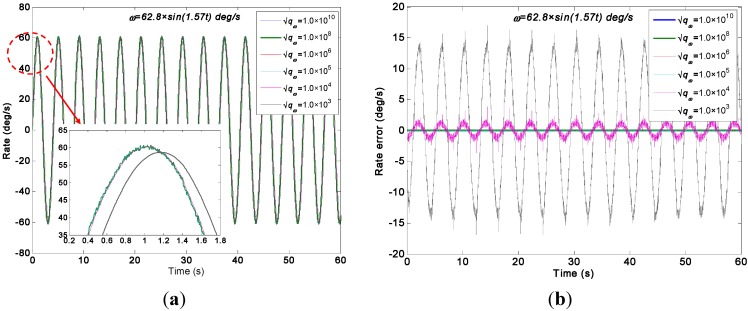
Test results of the virtual gyroscope by the direct estimated KF model with different values of $\sqrt{q_{\omega }}$ of a sinusoidal input signal [*ω =* 62.8 × *sin*(1.57*t*)°/s]. (**a**) Estimated rate signal; (**b**) Estimated errors.

**Figure 16:**
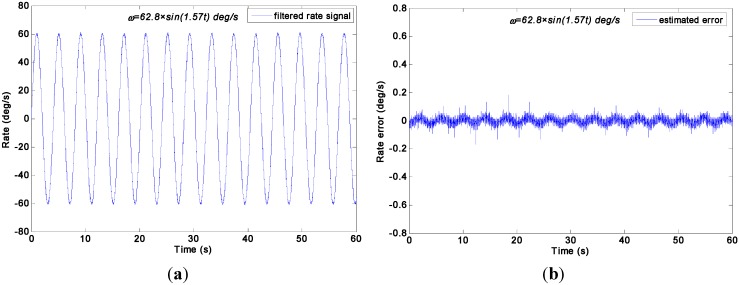
Test results of virtual gyroscope by the indirect differencing KF model of a sinusoidal input signal [*ω =* 62.8 × *sin*(1.57*t*)°/s]. (**a**) Estimated rate signal; (**b**) Estimated errors.

The swing rate signal tests were performed on a turntable, which was controlled to swing with an angle amplitude of *A =* 40° and a frequency of *f =* 0.25 Hz. Thus, the input signal is *ω =* 62.8 × *sin*(1.57*t*)°/s. The outputs of gyroscope signals were collected with a sampling rate of 200 Hz. The output signals of the gyroscope array are shown in Figure 14. With the choice $\sqrt{q_{\omega }}$ of 1.0 × 10^10^, 1.0 × 10^8^, 1.0 × 10^6^, 1.0 × 10^5^, 1.0 × 10^4^, and 1.0 × 10^3^°/h, the test results of the virtual gyroscope with two KF models are shown in Figure 15 and Figure 16 respectively. The detailed results are illustrated in Table 5.

Figure 15 shows that the estimated rate signal by the direct KF model can reflect and reproduce the dynamic property of the input signal while the $\sqrt{q_{\omega }}$ is chosen as 1.0 × 10^5^, 1.0 × 10^6^, 1.0 × 10^8^, and 1.0 × 10^10^°/h, and the amplitude of estimated rate signal reaches 61.3°/s. This value is basically in accordance with the input signal of 62.8°/s without a large attenuation. However, when the $\sqrt{q_{\omega }}$ was chosen as 1.0 × 10^4^ and 1.0 × 10^3^°/h, the estimated rate signal could not reproduce the dynamic property of the input signal, especially resulting in a severe amplitude attenuation and phase delay because, in such case, the KF bandwidth determined by the $\sqrt{q_{\omega }}$ cannot satisfy the requirement of accurately reflecting the dynamic property of the input rate signal. This point is consistent with the simulation analyses in Section 4.2, and the only difference is that the value of *q_ω_* obtained in the amplitude attenuation is different from the simulation. This variation is due to the different values of KF parameters in the simulation and experiment test. Concretely, there are two aspects that result in a different $\sqrt{q_{\omega }}$ for simulation and experiment test: (1) The ARW and RRW noises statistics of the ADXRS300 are different from the noises variance setting in the simulation; (2) The dynamic characteristic of the input rate signals are different from the simulation because of the various signal amplitude and frequency.

**Table 5:** Compared results of two virtual gyroscope KF models with a sinusoidal input rate signal [*ω =* 62.8 × *sin*(1.57*t*)°/s].

Virtual Gyroscope Model	$\sqrt{q_{\omega }}$ (°/h)	Amplitude of Rate Signal (°/s)	STD of Rate Error (1σ, °/s)
Direct Estimated Model	1.0 × 10^10^	61.2876	0.6195
	1.0 × 10^8^	61.2876	0.6011
	1.0 × 10^6^	61.2876	0.5428
	1.0 × 10^5^	61.2554	0.5202
	1.0 × 10^4^	60.6423	0.9140
	1.0 × 10^3^	58.7606	9.7288
Differencing Estimated Model	—	61.2876	0.6079
Original Individual Gyroscope	—	62.6428	1.6231

In particular, compared with the simulation in Section 4.2, which aimed to obtain an optimal rate signal estimate, the parameter *q_ω_* in the swing test is chosen with a larger value because the dynamic variation of input rate signal in the test is higher than that of the input rate signal in the simulation.

Table 5 indicates that the 1σ error of the individual gyroscopes at approximately 1.6231°/s was reduced to 0.5202°/s and 0.5428°/s by the direct estimated KF model with choice of $\sqrt{q_{\omega }}$ = 1.0 × 10^5^°/h and 1.0 × 10^6^°/h respectively, which made a reduction factor of about 3. By contrast, a reduction factor of about 2.6 was obtained by the differencing estimated KF model. Consequently, this finding demonstrates that the performances of the two KF models are comparable because of the high dynamic property of the input rate signal. Therefore, the simulation conclusion is clearly confirmed.

## 6. Conclusions

The performance of virtual gyroscope for the two different KF models was explored and analyzed in the dynamic condition. Simulations and experiments were carried out to quantify the accuracy of the combined rate signals. Six MEMS gyroscopes were utilized to develop a virtual gyroscope system. A gyroscope 1σ rate error was reduced from 1.4558°/s to 0.1203°/s by the direct estimated KF model in a constant rate test, which made a reduction factor of about 12.1, while the 1σ error by the differencing estimated KF model was 0.5974°/s. The findings also demonstrated that the estimated rate signal combined by two KF models could reflect the dynamic characteristic of the input rate signal in the swing rate test, and displayed a reduction factor of about 3 for the dynamic noise. Note that the choice of *q_ω_* should be based on the practical application environment, *i.e.*, the bandwidth of the input signal. One of the important aspects of the technology of MEMS gyroscope is how to adaptively tune the parameter *q_ω_* online. In the successive work, the technology of fuzzy logic inference system will be adopted to dynamically adjust the value of *q_ω_* by using of the variance of the input rate signal in a specific measurement period as the input variable for a fuzzy logic adaptive controller.

The performance of direct estimated KF model is, therefore, much higher than that of the differencing estimated KF model with a constant or lower input rate signal dynamic characteristic because the KF bandwidth of direct estimated KF model can be adjusted. Nevertheless, both KF models have a similar performance, while the input rate signal has a higher dynamic characteristic. Thus, if the input rate signal has a lower dynamic behavior, the direct estimated KF model should be selected to obtain a high accuracy improvement. This can provide a useful guidance to choose the KF model for the implementing system.

In addition, the noise correlation in the gyroscope array is the most important factor for a system to remarkably reduce noise and improve accuracy. The accuracy improvement will be much higher than that of the paper if there is a favorable correlation of component gyroscopes. This issue needs to be further studied in our future work.
